# The Effects of Amphetamine and Methamphetamine on the Release of Norepinephrine, Dopamine and Acetylcholine From the Brainstem Reticular Formation

**DOI:** 10.3389/fnana.2019.00048

**Published:** 2019-05-10

**Authors:** Michela Ferrucci, Fiona Limanaqi, Larisa Ryskalin, Francesca Biagioni, Carla L. Busceti, Francesco Fornai

**Affiliations:** ^1^Human Anatomy, Department of Translational Research and New Technologies in Medicine and Surgery, University of Pisa, Pisa, Italy; ^2^IRCCS Neuromed, Pozzilli, Italy

**Keywords:** methamphetamine, norepinephrine, brainstem reticular formation, addiction, arousal, neurotoxicity, hypertension

## Abstract

Amphetamine (AMPH) and methamphetamine (METH) are widely abused psychostimulants, which produce a variety of psychomotor, autonomic and neurotoxic effects. The behavioral and neurotoxic effects of both compounds (from now on defined as AMPHs) stem from a fair molecular and anatomical specificity for catecholamine-containing neurons, which are placed in the brainstem reticular formation (RF). In fact, the structural cross-affinity joined with the presence of shared molecular targets between AMPHs and catecholamine provides the basis for a quite selective recruitment of brainstem catecholamine neurons following AMPHs administration. A great amount of investigations, commentary manuscripts and books reported a pivotal role of mesencephalic dopamine (DA)-containing neurons in producing behavioral and neurotoxic effects of AMPHs. Instead, the present review article focuses on catecholamine reticular neurons of the low brainstem. In fact, these nuclei add on DA mesencephalic cells to mediate the effects of AMPHs. Among these, we also include two pontine cholinergic nuclei. Finally, we discuss the conundrum of a mixed neuronal population, which extends from the pons to the periaqueductal gray (PAG). In this way, a number of reticular nuclei beyond classic DA mesencephalic cells are considered to extend the scenario underlying the neurobiology of AMPHs abuse. The mechanistic approach followed here to describe the action of AMPHs within the RF is rooted on the fine anatomy of this region of the brainstem. This is exemplified by a few medullary catecholamine neurons, which play a pivotal role compared with the bulk of peripheral sympathetic neurons in sustaining most of the cardiovascular effects induced by AMPHs.

## Introduction

**Figure 1:**
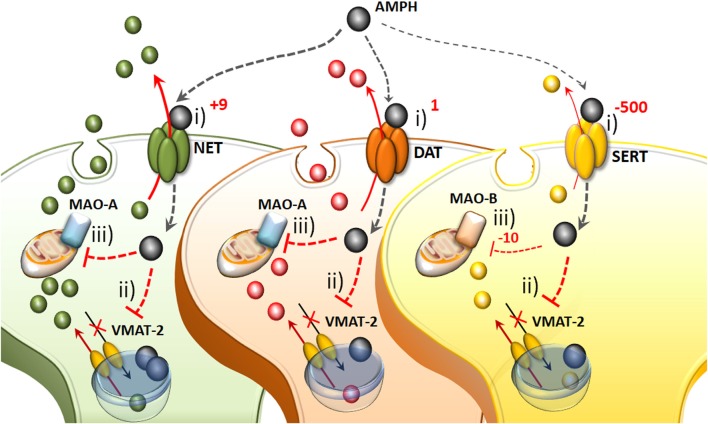
The molecular mechanisms of amphetamine(s) (AMPHs) in monoamine-containing neurons. (i) The primary molecular target, which provides neuronal selectivity for AMPHs, consists in the plasma membrane transporter. In fact, AMPHs behave as competitive substrates for the re-uptake through the NE transporter (NET), dopamine (DA) transporter (DAT) and 5-HT transporter (SERT; Rothman and Baumann, 2003; Fleckenstein et al., 2007). These transporters normally work by taking up extracellular monoamines to the axoplasm, which is the main mechanism to terminate their activity (Iversen et al., 1965; Axelrod and Kopin, 1969; Coyle and Axelrod, 1971; Aggarwal and Mortensen, 2017). Cross-affinity between AMPHs and neurotransmitters contributes to generate the quite selective storage of AMPHs within specific neurons. Once bound to the plasma membrane transporter, AMPHs enter the axoplasm while reverting the transport direction (Sulzer et al., 1993). This occurs mostly for catecholamine neurons since AMPHs strongly discriminate between SERT, to which they bind with much lower affinity (500-fold less) compared with DAT and NET (Rothman and Baumann, 2003). In particular, AMPHs bind to the NET with five-to-nine-fold higher affinity compared with the DAT (Rothman and Baumann, 2003). This is the main reason why AMPHs release NE more potently than DA and much more than 5-HT (Rothman et al., 2001). (ii) Within monoamine axons, AMPHs encounter a second specific target called vesicular monoamine transporter type-2 (VMAT-2), which is also shared with monoamines. In this way, AMPHs enter the synaptic vesicles. At this level, AMPHs impair the acidification of the vesicle, which generates an acidic pH (Sulzer and Rayport, 1990; Sulzer et al., 1993, 1995). This acidic environment is erased by AMPHs, which rise the vesicular pH value from 4 up to 7, which corresponds to a 1,000-fold increase in the concentration of H^+^ ions. Thus, monoamines, which are weak bases, are charged at low pH, while at a neutral pH lose their charge, and diffuse through the vesicle membrane, thus massively invading the axoplasm (Brown et al., 2000, 2002; Pothos et al., 2000). In this way, axonal monoamines either passively or *via* a reverted plasma membrane transporter fill extracellular space where they reach a massive concentration (Sulzer et al., 1995, 2005). (iii) The third molecular target, which is impaired by AMPHs, is the mitochondrial-bound enzyme monoamine oxidase (MAO). Both MAO-A/-B iso-enzymes oxidatively deaminate DA, NE and 5-HT. Nonetheless, MAO-A/-B isoforms differ in substrate preference, inhibitor affinity and regional distribution within either single neurons or different animal species (Robinson et al., 1977; Youdim, 1980; Sourkes, 1983; Gesi et al., 2001; Youdim et al., 2006; Bortolato et al., 2008). These differences are seminal to explain the specific effects of AMPHs within various monoamine neurons. In fact, MAO-A, are competitively inhibited by methamphetamine (METH) with a 10-fold higher affinity compared with MAO-B. MAO-A is placed within synaptic terminals of DA and NE neurons, while MAO-B are the only isoform operating within 5-HT terminals and non-catecholamine neurons. Thus, apart from rats and a few animal species, the effect of AMPHs on the amount of extracellular monoamines is remarkable concerning NE and DA, being less pronounced for 5-HT.

Amphetamine (AMPH) and mostly methamphetamine (METH) are widely abused psychostimulants, which possess a phenylethylamine structure. In the present review article, we focus on both compounds, which, from now on are referred to as AMPHs meant “*sensu stricto*” to rule out other amphetamine-related compounds. Both acute and chronic AMPHs intake/administration determines behavioral, purely motor, and vegetative alterations; each effect relies on a quite specific constellation of reticular nuclei, which often overlap. This calls for a constant reference to the functional anatomy of the brainstem reticular formation (RF), which stands as the seminal brain area to comprehend the neurobiology of AMPHs. Short-term effects of AMPHs include intense euphoria, increased heart rate, hypertension, hyperthermia, excitation, alertness and wakefulness (Meredith et al., 2005; Homer et al., 2008; Moratalla et al., 2017), which can be related to specific groups of brainstem nuclei. On the other hand, reiterated intake/administration of AMPHs produces long-lasting alterations, which may be the consequence of neurotoxicity or being produced by persistent epigenetic changes driving marked plastic phenomena in some brain areas (Battaglia et al., 2002; Robison and Nestler, 2011; Godino et al., 2015; Limanaqi et al., 2018). Behavioral alterations include motor and psychiatric effects such as hyper-locomotion, stereotypies, addiction, craving, aggressiveness, anorexia, psychosis, depression, cognitive impairments, and altered cortical excitability ranging from sleep alterations up to seizures (Meredith et al., 2005; Brown et al., 2011; Marshall and O’Dell, 2012; Moratalla et al., 2017). All these effects vary over time following reiterated exposure and some of them occur as the consequence of neurotoxicity or the onset of “neuronal sensitization” (Robinson and Berridge, 2000). In fact, when administered chronically and/or at high doses, AMPHs and mostly METH produce toxicity in specific brain regions or even in peripheral organs, especially those receiving dense innervation by the sympathetic nervous system (Liu and Varner, 1996; Albertson et al., 1999; Darke et al., 2008; Volkow et al., 2010; Thanos et al., 2016). Most of the effects induced by AMPHs are grounded on the powerful release of a variety of neurotransmitters, which occurs through a mechanism owning a fair anatomical specificity. The lateral column of the brainstem RF contains neuronal populations, which possess common molecular targets characterizing specific neuronal phenotypes. These very same targets are recruited quite selectively by AMPHs administration (Figure 1). In fact, the chemical structure of AMPHs is characterized by an aromatic ring and a nitrogen on the aryl side-chain (Biel and Bopp, 1978), which recapitulates most monoamine neurotransmitters including catecholamines (dopamine, DA, norepinephrine, NE, epinephrine, E) and indoleamines (serotonin, 5-HT, tryptamine and other trace amines; Heal et al., 2013). In the light of a cross affinity, AMPHs behave as a competitive substrate for monoamine transporters including NE transporter (NET), DA transporter (DAT) and 5-HT transporter (SERT; Rothman and Baumann, 2003; Fleckenstein et al., 2007; Sitte and Freissmuth, 2015). This is the reason why AMPHs target quite selectively those neurons, which produce monoamine as neurotransmitters. Such a simple concept explains why the RF is, at large, the target brain region for the mechanism of action of AMPHs. Likewise, we focus this review on this very same area to analyze in depth those effects not fully explained by DA neurons which are hosted in the high mesencephalic reticular nuclei. In detail, most monoamine-containing nuclei of the ponto-medullary RF are placed in a quite restricted brain region corresponding to the median and lateral column of the RF of the brainstem. The basis for both short- and long-term behavioral, motor and vegetative effects of AMPHs stems from such a selective uptake and common intracellular targets of AMPHs within these monoamine-containing neurons (Bucci et al., 2017). These neurons produce widespread innervation of a variety of limbic, motor and iso-cortical areas where ultimately the effects of AMPHs are produced. In fact, at sub-cellular levels, the targets of AMPHs are both the cell body and mostly, the axon terminal. The latter represents the primary AMPHs’ target. In fact, the powerful release of monoamines relies on the effects of AMPHs on axon varicosities, where monoamines are concentrated in baseline conditions. AMPHs massively release neurotransmitters in high amount, which can be quantitatively assessed by placing microdialysis probes within all brain regions innervated by monoamine neurons. At the same time, this can be correlated with a number of systemic effects, which are mediated by each brain region under a powerful reticular monoamine innervation. In fact, following AMPHs there are dramatic changes in breathing, blood pressure, locomotor activity, muscle tone, sleep-wake cycle, mood, orienting to novelty, arousal, anxiety, reward along with innumerous vegetative and somatic phenomena occurring in the human body. A great amount of investigations, commentary manuscripts and books focused on the effects of AMPHs abuse on DA-containing neurons within the mesencephalon (Seiden et al., 1976; Wagner et al., 1979; Nielsen et al., 1983; Di Chiara and Imperato, 1988; Sonsalla et al., 1989; Cadet et al., 1994; Delle Donne and Sonsalla, 1994; Fornai et al., 1997, 2001; Battaglia et al., 2002; Ferrucci et al., 2008; Moratalla et al., 2017). Instead, the contribution of low brainstem nuclei is much less investigated and, apart from the Locus Coeruleus (LC), only a few manuscripts investigated the recruitment of the variety of catecholamine neurons within the low brainstem during AMPHs administration. Despite not being widely investigated, this point deserves very much attention since the specific functional anatomy of these nuclei may lead to comprehension of the brainstem-related nature of a variety of AMPHs-induced alterations. Therefore, in the present review article, we avoid the analysis of DA neurons of the Substantia Nigra pars compacta (SNpc) and Ventral Tegmental Area (VTA), and focus instead on catecholamine nuclei of the low brainstem. In particular, we focus on NE nuclei located within the lateral column of the bulbo-pontine RF and a mixed neuronal population within the dorsal raphe/periaqueductal gray (PAG), which contains a subset of NE and DA neurons (Battenberg and Bloom, 1975; Saavedra et al., 1976; Steinbusch et al., 1981; Nieuwenhuys et al., 1988; Baker et al., 1990, 1991; Lu et al., 2006; Li et al., 2016; Bucci et al., 2017; Cho et al., 2017). We also overview cholinergic cells placed in the lateral column of the pontine RF, which extend up to the lateral wings of the dorsal raphe (Satoh et al., 1983; Nieuwenhuys et al., 2007; Vasudeva and Waterhouse, 2014; de Oliveira et al., 2016). In detail, the dorsal raphe contains 5-HT neurons in all nuclear regions, while catecholamine and acetylcholine cells are placed in the most rostral extent of the dorsal raphe both in rodents and humans (Saavedra et al., 1976; Steinbusch et al., 1981; Baker et al., 1990, 1991; Nieuwenhuys et al., 2007; Mai and Paxinos, 2012; Li et al., 2016; Cho et al., 2017). As shown by pioneer studies, the amount of NE neurons in the dorsal raphe is remarkable, being about one-third of 5-HT neurons. Instead, the amount of DA cells is roughly a half compared with NE neurons, at least in the rat (Saavedra et al., 1976). While the median ventral PAG contains a few 5-HT neurons, both NE and DA neurons are placed in the ventral/ventrolateral corner of the PAG (Hökfelt et al., 1984; Dougalis et al., 2012; Mai and Paxinos, 2012). DA cells occurring within this brain region are known as A10dc (dorsal central) in order to distinguish them from the adjacent VTA DA neurons (Hökfelt et al., 1984). Again, NE neurons placed at this level represent the dorsal and rostral extent of the LC complex. This rostral extent was described in humans as nucleus epicoeruleus by Mai and Paxinos (2012). In addition to the presence of NE-containing neurons, the dorsal raphe receives a powerful NE innervation. This is supported by findings in humans, where the amount of NET in the ventral nuclei of the dorsal raphe matches the amount of NET which can be measured within the LC (Ordway et al., 1997). There is a remarkable affinity of AMPHs for NET compared with DAT and SERT. In fact, AMPHs administration produces a more powerful release of NE compared with DA (Rothman et al., 2001; Weinshenker and Schroeder, 2007; Schmidt and Weinshenker, 2014). This means that behavioral effects induced by AMPHs in various animal species including humans can be partly explained by massive NE release (Rothman et al., 2001; Weinshenker et al., 2002; Weinshenker and Schroeder, 2007). Thus, despite a DA solely-based perspective about AMPHs and behavior, NE creeps back in participating to AMPHs-induced behavioral changes. This calls for dissecting further anatomical areas within NE-containing brainstem nuclei to comprehend the effects induced by AMPHs, along with considering the anatomical connections linking the bulbo-pontine with the mesencephalic RF as a biological substrate, which may sustain a key circuitry mediating the effects of AMPHs.

## The Functional Anatomy of the Catecholamine Reticular Nuclei of the Brainstem in the Effects of AMPHs

Since the present review is an attempt to relate the effects of AMPHs with specific NE nuclei of the brainstem, a preliminary synthetic overview of the neuroanatomy of these nuclei appears to be mandatory. This will make it easier to orient within the brainstem when referring to the site-specificity of the effects induced by AMPHs.

### NE-Containing Reticular Nuclei

**Figure 2:**
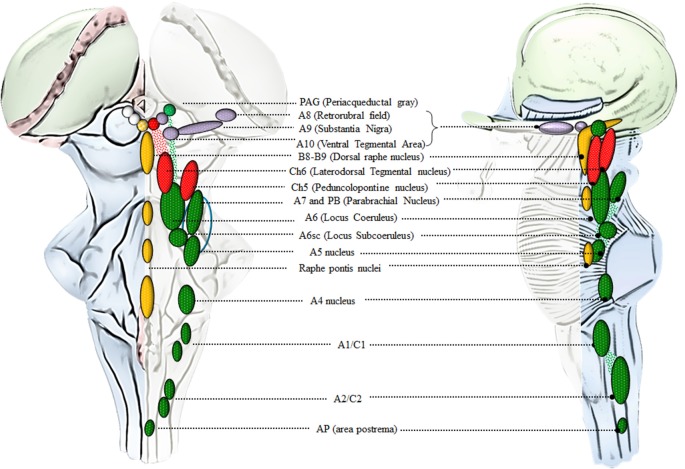
An anatomical overview of the brainstem reticular formation (RF). This cartoon provides a synthetic overview of the neuroanatomy of brainstem RF nuclei in order to foster orientation within the brainstem when referring to the site-specificity of the effects induced by AMPHs. Monoamine-containing nuclei are placed in a quite restricted brain region corresponding to the median and lateral column of the brainstem RF. In detail, the median column hosts 5-HT-containing nuclei (yellow), which extend along the medulla and pons to reach the mesencephalon at the level of the periaqueductal gray (PAG). 5-HT containing nuclei at this level represent the rostral extent of the dorsal raphe nucleus (B8-B9). Since an analysis of 5-HT neurons in the effects of AMPHs is beyond the scope of the present review article, we focus on the dorsal raphe nucleus just concerning the PAG region, which contains catecholamine neurons. In fact, the PAG hosts a mixed population of catecholamine and acetylcholine (ACh)-containing nuclei which are placed in the lateral column of the RF. This is the case of DA (violet), NE (green) and ACh (red) nuclei which represent the rostral extent of A10 Ventral Tegmental Area (VTA), A6 (LC) and CH6, respectively. Moving downstream to the mesencephalic DA nuclei (A8, A9, A10), a constellation of nuclei appears within the lateral column of RF. These include the two pontine ACh nuclei (Ch5 and Ch6) and a number of NE nuclei placed along the ponto-medullary RF, namely A6 (LC), A6sc (subcoeruleus), A7 which intermingles with NE neurons of the rostral PB nucleus (here represented as a continuum), A5, A4, and two mixed NE/E-containing groups in the medulla, namely A1/C1 [rostral ventrolateral medulla (RVLM)] and A2/C2 (dorsomedial cell group), which extends towards the obex within the AP (area postrema). The A7 neurons together with PB-NE neurons and the A5 nucleus contribute to define the Kolliker-Fuse nucleus (blue circle, Milner et al., 1986; Byrum and Guyenet, 1987). The A6 nucleus together with the A6sc, the medial PB in the rostral pons and the epicoeruleus nucleus within the PAG, constitute the LC complex.

Catecholamine-containing nuclei are mainly housed within the lateral extent of the RF (Figure 2). A very recent original manuscript provided stereological morphometry data encompassing all brainstem reticular catecholamine nuclei at one glance (Bucci et al., 2017). These include NE neurons of the pons and medulla, which were identified by using TH immunostaining (Bucci et al., 2017). For this reason, we will refer to NE-containing nuclei and we will include the E-related sub-nuclei as a putative attribute, since most of them are believed to represent a continuum with NE areas. This is the case of nuclear complexes known as A/C nuclear groups, where the letter “A” indicates NE neurons and the letter “C” indicates E neurons (Hökfelt et al., 1974). The A1/C1 cell group is placed in the sub-pial aspect of the rostral ventrolateral medulla (RVLM). The A2/C2, also known as dorsomedial cell group appears medially on the floor of the IV ventricle. Reticular neurons of A2/C2 intermingle with neurons of the dorsal nucleus of the vagus (DMV) and nucleus of the solitary tract (NTS) to constitute an overlapped, neuromelanin pigmentated area, which is named ala cinerea. The posterior region of ala cinerea extends towards the obex to constitute the area postrema (AP), which corresponds approximately to the chemoreceptor trigger zone (CTZ; Potes et al., 2010). A3/C3 area is still poorly investigated due to species variability (Howe et al., 1980; Vincent, 1988; Paxinos et al., 1995; Menuet et al., 2014). Similarly, fragmentary information deals with the A4 nucleus once believed to occur only in primates though it was recently identified in rodents (Bucci et al., 2017). The A5 nucleus is placed ventrally in the pons, close to the roots of the facial nerve. Moving towards the dorsal and medial aspect of the pons, these neurons form a continuum with other NE neurons belonging to the A6sc (nucleus subcoeruleus) and A6 (locus coeruleus, LC) nuclei. A5 and A6 (LC) represent the primary sources of NE afferents to the VTA and A1/C1 (Bucci et al., 2017). The A7 nucleus (lateral lemniscus nucleus) is placed in the pons, immediately lateral to the rostral end of the parabrachial (PB) nucleus. A6 (LC) is the biggest NE-containing nucleus within the central nervous system (CNS) and it is located in the upper part of the floor of the IV ventricle, within the pons. NE-containing neurons of LC, together with A6sc and the scattered TH-positive cells within the medial PB form a tube-shaped continuum, which is named LC complex. The A4 area, when present, can be considered within this complex as well (Bucci et al., 2017). In humans, the LC complex also includes the nucleus epicoeruleus, which occurs in the rostral dorsal raphe, within the ventrolateral PAG (Mai and Paxinos, 2012).

**Figure 3:**
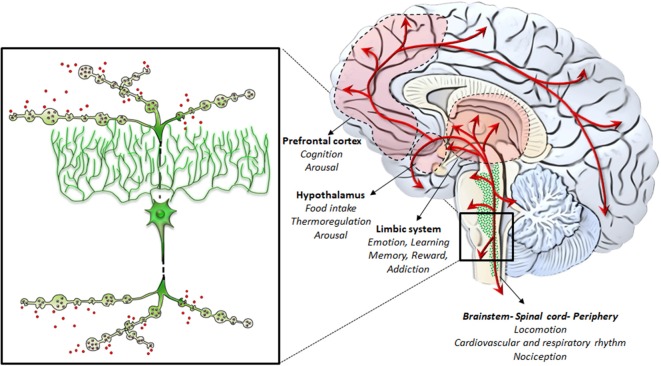
NE-containing neurons of the RF: from gross anatomy to the fine iso-dendritic nature. NE-containing neurons of the RF profusely branch their axons to release NE throughout the whole central nervous system (CNS) and the autonomic nervous system. This is grounded on the typical fine neuroanatomy of NE fibers. In fact, apart from the marked spreading of axonal projections due to a profuse collateralization (which is typical for neurons forming the iso-dendritic core of the RF), axon collaterals are gifted with varicosities, also named “boutons en passage.” This is key in the case of AMPHs, which promote massive NE release while impairing NE uptake. This allows NE to be released in the extracellular medium from each varicosity along the course of axon fibers to produce extra-synaptic, paracrine effects, even remotely from the release site *via* volume transmission. Altogether, these features synergize to produce widespread extra-synaptic effects following AMPHs-induced NE release in each brain area. This explains why AMPHs activate wide brain areas leading at the same time to different behavioral, motor and vegetative effects. These include the limbic system, the hypothalamus, the prefrontal cortex, as well as the whole brainstem, spinal cord and peripheral organs receiving NE sympathetic innervation.

Neurons belonging to the LC region profusely send their axons to the entire CNS, providing the main source of NE to the brain, and mostly, to the whole cerebral cortex (Loughlin et al., 1982). In addition, the fine neuroanatomy of NE (and catecholamine) fibers possesses typical features. In fact, apart from the marked spreading of axonal projections due to profuse collateralization, which is typical for neurons forming the isodendritic core of the RF, axon collaterals are characterized by the presence of varicosities, named “boutons en passage” (Figure 3). Since once released following AMPHs, NE persists in the extracellular space for a considerable amount of time until being taken back up by the NE terminal, the time persistency and the volume filled by AMPHs-induced extracellular NE are noticeable. In fact, AMPHs promote NE release and impair NE uptake. This allows NE to be released in the extracellular medium from each varicosity along the course of axon fibers to produce extra-synaptic, paracrine effects, even distant from the release site *via* a volume transmission. Altogether, these features synergize to produce extremely widespread effects following AMPHs activation of NE nuclei.

### The Role of NE Nuclei in AMPHs-Induced Behavioral Effects

**Figure 4:**
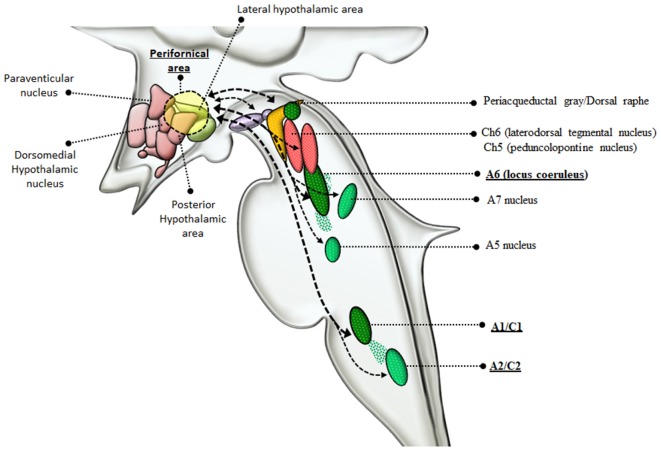
Connections between reticular nuclei and orexin-containing perifornical neurons of the hypothalamus. Orexin-containing neurons are placed within the dorsolateral hypothalamus corresponding to the perifornical area. Orexin perifornical neurons are profusely connected with a number of brainstem RF nuclei including the PAG/dorsal raphe, VTA, Ch6, Ch5, A6, A5, A7, A1/C1, A2/C2 (Marcus et al., 2001; Sharf et al., 2010; Mahler et al., 2012). However, among these neuronal groups, orexins display the highest neuro-anatomical and pharmacological specificity with NE-containing neurons, as witnessed by the strong reciprocal connections with LC and A1/C1 nuclei (thick dashed connectors) and the abundance of orexin receptors in these nuclei (McCabe and Leibowitz, 1984; van den Pol et al., 2002; Bayer et al., 2005; Gompf and Aston-Jones, 2008; Li et al., 2015; Ritter, 2017). In the light of such connections with NE neurons, orexin-containing neurons are dragged in a variety of functions including, arousal, locomotion, feeding, reward, sensitization, motivation (Sakurai et al., 1998; Sakurai, 2007; Sakurai and Mieda, 2011). Since AMPHs are powerful NE releasers and they strongly activate orexin-producing neurons as well, it is likely that the strong connections between orexin and NE-neurons of the LC and A1/C1 are implicated in the effects induced by AMPHs.

NE is key in mediating behavioral correlates effects of AMPHs. Specifically, NE sourcing from the RF strongly modulates AMPHs-induced behaviors and reward, which is the pre-requisite for sustaining reinforcing behavior leading to AMPHs addiction. In fact, reiteration of drug intake induces plastic changes within specific neuronal systems, which are necessary to develop craving and addiction (Pierce and Kumaresan, 2006). In this paragraph, specific reticular NE nuclei will be related to rewarding effects induced by AMPHs. From a general viewpoint, NE mediates a large amount of the rewarding effects induced by AMPHs as shown early in the ‘60s (Poschel and Ninteman, 1963; Stein, 1964; Sofuoglu and Sewell, 2009). This occurred in the context of the “Catecholamine theory of reward” (Hanson, 1966; Crow et al., 1972; Crow, 1972, 1973; Wise, 1978), where NE was thought to be the main neurotransmitter to produce reward following a variety of psychostimulants. In line with this, drugs reducing NE activity (by depleting NE stores, or inhibiting NE synthesis, or damaging NE axons) were shown to dampen intracranial self-stimulation (ICSS), which was used as an experimental model for reward (Fibiger and Phillips, 1974). A more detailed knowledge about the anatomy of the low brainstem RF provided the substrate to confirm the contribution of NE nuclei to the neurobiology of reward induced by psychostimulants (Wise, 1978; Ordway et al., 1997; Weinshenker and Schroeder, 2007). For instance, positive self-stimulation sites exist in the LC (A6) as well as along ascending NE pathways. These include: (i) the ventral NE bundle (VNB, which originates from the LC) as well as; (ii) the dorsal NE bundle (DNB); and (iii) the medial forebrain bundle (MFB), which originate from a number of NE nuclei within the pons and medulla, including LC, PB, A1 and A2 (Dresse, 1966; Stein and Wise, 1969; Crow et al., 1972; Wise et al., 1973; Ritter and Stein, 1974; Carlezon and Chartoff, 2007). In fact, apart from LC, caudal NE nuclei such as the A1/C1 and A2/C2 cell groups project profusely to the forebrain and hypothalamus (Nieuwenhuys et al., 1982; Mai et al., 2008; Berridge et al., 2012). As shown by retrograde tracing studies, the LC provides nearly 50% of NE input to the forebrain and hypothalamus, while A1/C1 and A2/C2 groups contribute from 25% to 40% (España and Berridge, 2006). This partly explains why AMPHs promote arousal (Berridge et al., 1999; Berridge and Stalnaker, 2002; Berridge, 2006). AMPHs-induced cortical arousal *via* NE release from these nuclei is associated with drug-seeking behavior and relapse (España et al., 2016). This may occur also *via* NE acting on hypothalamic perifornical orexin neurons. Orexins are hypothalamic neuropeptides implicated in a variety of behaviors including sleep/wakefulness, feeding and reward (Sakurai et al., 1998; Sakurai, 2007; Sakurai and Mieda, 2011). Recent studies suggest that orexins play a role in drug-induced sensitization and drug-seeking motivation, which occurs through a fair neuro-anatomical specificity (Sharf et al., 2010; Mahler et al., 2012). The effects of orexin-containing nuclei are grounded on their strong connection with reticular NE nuclei (Figure 4). In fact, NE nuclei of the brainstem represent the most densely orexin-innervated neuronal population (Date et al., 1999; Nambu et al., 1999; Peyron et al., 2000; Marcus et al., 2001). This occurs mostly within NE LC neurons, where orexin type 1 receptors (OX1R) are densely expressed to promote arousal and locomotor activity (Hagan et al., 1999; Marcus et al., 2001). A reciprocal modulation occurs between LC-NE and hypothalamic perifornical orexin-containing neurons (van den Pol et al., 2002; Bayer et al., 2005; Gompf and Aston-Jones, 2008). This is key in the case of METH, which increases orexin levels in METH abusers (Chen et al., 2016). Likewise, METH administration strongly activates orexin-producing neurons, as shown by the increase in c-Fos expression (Estabrooke et al., 2001; Cornish et al., 2012). A considerable amount of orexin receptors is present also in lower reticular NE groups including A1/C1 and A2/C2 (Marcus et al., 2001), which explains functional studies addressing the role of such a connection in food intake and AMPHs-induced anorexia (McCabe and Leibowitz, 1984; Li et al., 2015; Ritter, 2017). Apart from providing molecular and anatomical specificity, NE neurons are also involved in genetic susceptibility to AMPHs-induced behavioral effects. In fact, a genetic polymorphism affecting the limiting enzyme for NE synthesis, DA beta-hydroxylase (DBH), is involved in substance abuse disorders (Kalayasiri et al., 2014). Such a hypothesis was tested specifically in DBH KO mice, where AMPH was much more effective in producing DA-release and behavioral sensitization (Weinshenker et al., 2008). It is remarkable that such a potentiation of DA-release and behavioral sensitization is reminiscent of what occurs in LC-damaged mice following METH administration.

### Molecular Mechanisms Bridging Reticular NE Nuclei to AMPHs-Induced Behavioral Effects

This paragraph adds on the well established evidence that AMPHs-induced DA release is key to produce locomotor stimulation, sensitization and neurotoxicity to encompass the synergistic role of NE reticular nuclei in sustaining these effects. In fact, the powerful NE release contributes to AMPHs-induced hyper-locomotion and stereotypies. Stereotypies, which occur upon reiterated AMPHs administration increase progressively following the same dose of AMPHs as a typical expression of AMPHs-induced behavioral sensitization. This is achieved by multiple mechanisms, which include the stimulation of alpha1-adrenergic receptors (α1-ARs), as demonstrated since the early ‘80s (Dickinson et al., 1988; Drouin et al., 2002a,b; Vanderschuren et al., 2003). Consistently, reduced hyper-locomotion and a loss of sensitization occur after α1B-AR inhibition or in mice genetically lacking this receptor subtype (Auclair et al., 2002, 2004). Consistently we found that α1B-AR-KO mice are also protected from METH-induced toxicity (Battaglia et al., 2003). This confirms data from Zuscik et al. (2000) who showed that overexpression of α1B-ARs in mice leads to an extended degeneration, which appears reminiscent of METH-induced neuronal damage (Fornai et al., 2004) and multiple system atrophy (Zuscik et al., 2000). These mice also develop spontaneous seizures, which are a typical effect observed upon METH-induced sensitization. Remarkably, when we used α1B-AR-KO mice, seizures were prevented (Pizzanelli et al., 2009). Again, activation of α1B ARs accounts for the increase in burst firing of midbrain DA neurons induced by AMPHs (Pan et al., 1996; Paladini et al., 2001), while α1B ARs antagonists suppress DA-related behaviors stimulated by AMPHs (Poncelet et al., 1983; Snoddy and Tessel, 1985; Tessel and Barrett, 1986; Dickinson et al., 1988; Mavridis et al., 1991; Blanc et al., 1994). Thus, α1B-ARs play a strong role in the deleterious effects induced by METH in the brain. In contrast, the peripheral effects induced by METH on NE neurons appear to rely on α1A-ARs since no effects are determined by α1B-ARs (Kikuchi-Utsumi et al., 2013).

Consistently, the selective inhibition of α1-AR within the nucleus accumbens (NAc) or prefrontal cortex abolishes hyper-locomotion induced by AMPHs (Blanc et al., 1994; Darracq et al., 1998). Again, amplification of AMPHs-induced locomotor activity occurs by increasing extracellular levels of NE *via* NET inhibition or by enhancing NE release *via* blockade of inhibitory pre-synaptic α2-ARs (Dickinson et al., 1988; Xu et al., 2000; Juhila et al., 2005). Data concerning the effects of the pharmacological modulation of NE receptors on AMPHs-induced behavior are mostly related to NE released from LC neurons. Thus, LC-NE activity appears to be crucial for sensitizing AMPHs-induced behavior and toxicity, although other nuclei need to be investigated more extensively. For instance, catecholamine neurons of the AP are relevant, since a damage to this area increases locomotor activity while facilitating stereotypies (Costall et al., 1981). In addition, A1/C1 and, to a lesser extent, A2/C2 neurons, which are connected with orexin-containing perifornical neurons of the hypothalamus (Figure 4), modulate food intake and contribute to anorexia induced by AMPHs (McCabe and Leibowitz, 1984; Li et al., 2015; Ritter, 2017). The A5 nucleus sends descending axons to the spinal cord down to the lumbosacral tract (Westlund et al., 1981). A5- together with A7-neurons are involved in anti-nociceptive effects, and they are likely to mediate AMPHs-dependent analgesia (Proudfit, 1988; Miller and Proudfit, 1990). AMPHs also target the PB, a critical integrative site within the brainstem being involved in pain, satiety, taste, arousal, breathing and blood pressure (Hajnal et al., 2009; Martelli et al., 2013; Davern, 2014). The involvement of PB nucleus was investigated independently of NE release only in the context of conditioned taste aversion induced by AMPHs (Krivanek, 1997). In fact, within PB, AMPHs increase protein kinase C (PKC) activity, placed downstream to METH-induced signaling and toxicity (Lin et al., 2012).

### The Brainstem NE Nuclei and AMPHs-Induced Autonomic Effects

**Figure 5:**
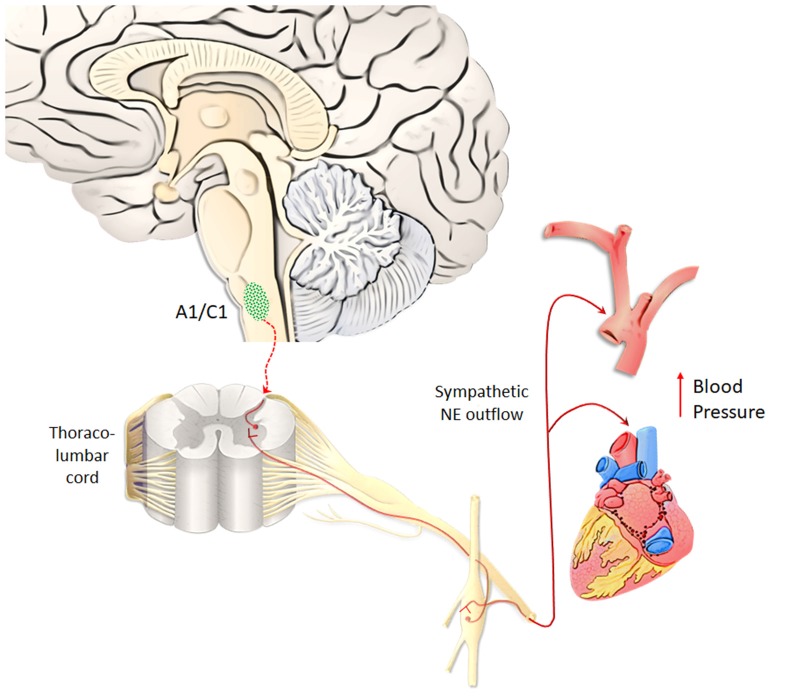
A1/C1 neurons as a key center to control blood pressure. Hypertension produced by AMPHs largely depends on A1/C1 medullary neurons, which regulate the vascular tone, heart rate and blood pressure. NE released from A1/C1 neurons regulates blood pressure by directly activating sympathetic pre-ganglionic neurons (SPNs), which in turn stimulate the heart and produce contraction of smooth muscle within blood vessels. Due to a powerful NE-release by AMPHs, these peripheral targets innervated by the A1/C1 complex are strongly activated during AMPHs administration (Liu and Varner, 1996). This response occurs in a sensitized manner following repeated dosing of AMPHs (Marchese et al., 2017).

A1/C1 neurons are anatomically organized in a roughly viscerotopic manner, in order to allow specific subsets of cells to control different visceral functions, encompassing circulation, breathing, glycemia, inflammation (Guyenet et al., 2013). A1/C1 neurons are mostly involved in regulating blood pressure (Reis et al., 1984). In fact, apart from activating pre-ganglionic vasomotor neurons, A1/C1 neurons control vasopressin release and sodium/water balance (Blessing and Willoughby, 1985; Guyenet, 2006). Thus, due to a powerful NE-release by AMPHs, it is expected that all regions being innervated by the A1/C1 complex will be activated during AMPHs administration. This may explain why AMPHs induce a severe increase in blood pressure (Liu and Varner, 1996), which was once believed to be solely due to peripheral NE. Thus, quite selective effects of AMPHs on a discrete neuron number of the medulla are supposed to provide a generalized increase in blood pressure (Figure 5). This key catecholamine nucleus adds on and surpasses the whole peripheral NE system in mediating AMPHs-induced hypertension. Thus, hypertension produced by AMPHs largely depends on a few central neurons, which regulate the vascular tone. Similarly to behavioral effects, the visceral responses to AMPHs are characterized by sensitization. Remarkably, even a single dose of METH may induce a sensitized response in blood pressure, which is accompanied by increased c-Fos immunoreactivity within TH-positive neurons of A1/C1 area and LC (Marchese et al., 2017). These findings confirm an overlap between behavioral and vegetative effects induced by AMPHs. In sharp contrast with a severe increase in blood pressure, chronic METH may lead to sudden and severe hypotension with bradycardia, which may lead to a lethal cardiovascular collapse (Chan et al., 1994; Ago et al., 2006; Miyashita et al., 2007). This is also related to a direct effect of METH within A1/C1 area, which mediates METH-induced increase in heart rate and arterial pressure (Liu and Varner, 1996), while at high and/or reiterated doses, METH may produce a selective neuronal death of A1/C1 (Li et al., 2012). This neuronal loss abolishes the descending activation of sympathetic pre-ganglionic neurons (SPNs), which are no longer able to stimulate the heart and produce contraction of smooth muscle within the blood vessels. This causes a sudden fall in blood pressure leading to METH-induced cardiovascular collapse (Li et al., 2012). These data are relevant to understand the key role of the reticular nuclei in regulating blood pressure while disclosing a previously overlooked neurotoxicity of METH on central NE neurons. In fact, these data demonstrate that NE neurons, apart from modulating METH toxicity to DA cells (Fornai et al., 1995, 1996a,b, 1997, 1998, 1999; Weinshenker et al., 2008), may also represent a primary target of METH toxicity. In fact, the A1/C1 nuclei innervate the SNPs being the pivot to provide direct excitatory input to the thoraco-lumbar sympathetic column of the cord (Ross et al., 1984). These neurons represent a critical link between the central respiratory rhythm generator and the vasomotor outflow (Guyenet et al., 1990). A multi-faceted signaling mechanism between A1/C1 cell groups and SPNs in the spinal cord is witnessed by several neuropeptides (such as prolactin, substance P, and cocaine- and amphetamine-regulated transcript, Cart, peptide), which are co-released within target areas (Chen et al., 1999; Dun et al., 2002). It is remarkable that METH persistently increases the expression of Cart peptide *via* epigenetic mechanisms (Jayanthi et al., 2017), which suggest that in addition to NE itself, neuropeptides produced by reticular NE neurons play a role in AMPHs-induced autonomic alterations. This is not surprising since NE nuclei are widely involved in neural circuitries, which regulate both behavioral and autonomic effects induced by AMPHs. For instance, A1/C1 sends visceral information to LC (Aston-Jones et al., 1986, 1991; Guyenet, 1991), which in turn, projects to midbrain DA neurons (Kirouac and Ciriello, 1997; Mejías-Aponte et al., 2009). In this way, DA neurons are recruited by these neurons, which mediate visceral effects produced by AMPHs. Such an integrated scenario indicates that behavioral and vegetative effects produced by AMPHs, despite being primarily processed within different nuclei, then converge in a common circuitry, which encompasses most catecholamine containing nuclei of the brainstem RF. In this way, depending on which nucleus we focus on, different effects produced by AMPHs can be mechanistically explained by the specific neuro-anatomical connections of this very same nucleus. This is not surprising at all when considering the simultaneous activation of the peripheral NE sympathetic nervous system when a demanding environmental task is activating the NE ascending reticular nuclei to produce arousal or during rewarding stimuli.

## The Interplay Between NE and DA in AMPHs-Induced Behavior

**Figure 6:**
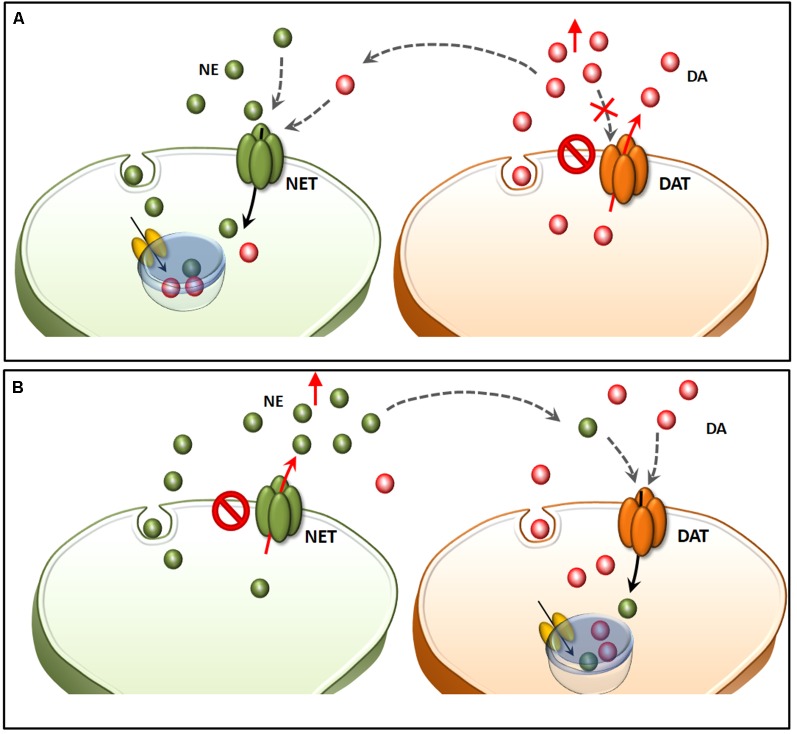
Structural similarities between NET and DAT confound neuron-specific NE and DA compartmentalization. Both NET and DAT represent the evolutionary divergence of a single catecholamine transporter (meNET), which was isolated and characterized already in the brain of the teleost fish medaka (Roubert et al., 2001). In the light of a strong structural similarity, both NET and DAT take up extracellular DA with a similar potency **(A)**. In fact, in the presence of an excess of extracellular DA (due to either a selective blockade of DAT or a reverted direction of DA transport), this may compete effectively with NE, thus being inappropriately stored within NE terminals. This explains why NE axons may internalize DA in the absence of DAT (Rocha et al., 1998). This same phenomenon also explains why in some instances selective NET inhibitors may paradoxically increase extracellular DA **(B)**. In fact, when a powerful NE release occurs in a densely DA-innervated area, it is very likely that extracellular NE is taken up mostly by fraudulent DA axons instead of authentic NE terminals.

Within a context of NE-dependent reward, a balanced dual perspective indicates that AMPHs need to converge on both DA and NE cells in order to be fully effective in producing reward. This was already hypothesized in a pioneer manuscript by Fibiger and Phillips (1974). In fact, caudal nuclei of the RF strongly connects with midbrain reticular DA nuclei. This circuitry originated during phylogeny, as biochemical, anatomical and physiological features strongly witness for an evolutionary continuum between mesencephalic DA neurons and more caudal NE cell groups (Bucci et al., 2017). In fact, profuse and reciprocal connections establish between NE/E nuclei within the pons and medulla and midbrain DA nuclei (Simon et al., 1979; Deutch et al., 1986; Grenhoff and Svensson, 1993; Grenhoff et al., 1993; Liprando et al., 2004; Mejías-Aponte et al., 2009). For instance, DA neurons of the Retrorubral Field (RRF, A8) and VTA (A10) receive abundant NE innervation, which is provided mainly from the nuclei A1, A2, A5 and A6 (Mejías-Aponte et al., 2009). Thus, DA and NE systems do not represent separate compartments within the CNS but rather an interconnected system, which share key neurobiological features making it as the endogenous circuitry where AMPHs electively impinge to produce a number of systemic effects. The strong anatomical connections between NE and DA systems are conserved at molecular level. This is best represented by the phylogeny of NET and DAT, which indeed represent the evolutionary divergence of an archaic single catecholamine transporter (meNET), which was isolated and characterized already in the brain of the teleost fish medaka (Roubert et al., 2001). This ancestral carrier is very similar to both the human NET and DAT, showing 70% and 64% amino acid homology, respectively. In fact, NET responds to AMPHs similarly to DAT and it represents the main gateway for AMPHs to invade NE terminals and to reach specific sub-cellular and molecular targets (Seidel et al., 2005). For instance, following AMPHs administration there is a down-regulation of NET and DAT, which are both stored in endosomes (Annamalai et al., 2010; Hong and Amara, 2013). Following AMPHs both DAT and NET, which remain on the plasma membrane, revert their transport of catecholamine (Sulzer et al., 1993; Robertson et al., 2009). Moreover, both transporters tend to be internalized within the terminals once bound to AMPHs. As mentioned above there is a strong similarity between NET and DAT. In fact, they are able to take up extracellular DA with a similar potency (Rothman et al., 2001). This affinity may confound the neuron-specific compartmentalization (Figure 6). In the presence of an excess of extracellular DA, this may compete effectively with NE, thus being inappropriately stored within NE terminals (Amara and Kuhar, 1993; Ramamoorthy et al., 2011; Borgkvist et al., 2012). This explains why in some instances selective NET inhibitors may paradoxically increase extracellular DA (Reith et al., 1997), while NE axons may internalize DA in the absence of DAT (Rocha et al., 1998). Again, when a powerful NE release occurs in a densely DA-innervated area, it is very likely that extracellular NE is taken up mostly by fraudulent DA axons instead of authentic NE terminals (Figure 6). This needs to be taken into account when considering the effects of AMPHs, since the fine structure of a given brain region may switch considerably the ratio of a combined mechanism of action upon both DA and NE systems. This fully applies to the different brain areas which sustain the reinforcing and rewarding effects of AMPHs (the richly DA-innervated NAc compared with densely NE-enriched allo-cortical regions).

## The Molecular Mechanisms of Brainstem DA-NE Interplay in the Behavioral Effects Induced by AMPHs

NE sourced by the reticular nuclei of the low brainstem is key for AMPHs-induced behavior (Rothman et al., 2001; Weinshenker and Schroeder, 2007; Weinshenker et al., 2008). This occurs also *via* amplification of DA-related rewarding and reinforcing properties. This is not surprising given the profuse reciprocal connections between NE and DA nuclei. In particular, LC innervates almost all brain areas, which receive DA innervation throughout the mesolimbic and mesocortical systems, including the ventral striatum and the prefrontal cortex (Nicola and Malenka, 1998). In fact, NE axons from LC neurons regulate DA release in the prefrontal cortex (Gresch et al., 1995; Devoto et al., 2005), while a damage to LC projections with DSP-4 alters baseline or stimulated DA release in the NAc (Lategan et al., 1992). This is consistent with studies showing that depletion of NE in the prefrontal cortex potentiates AMPHs-induced behavioral sensitization through striatal DA release (Ventura et al., 2003). In addition to α1B ARs involvement in DA-related behaviors and toxicity induced by AMPHs (Poncelet et al., 1983; Snoddy and Tessel, 1985; Tessel and Barrett, 1986; Dickinson et al., 1988; Mavridis et al., 1991; Blanc et al., 1994), the role of β-ARs has been investigated as well. In fact, Albers and Sonsalla (1955) showed that a β-AR blocker prevents AMPHs-induced DA toxicity, and a subsequent study confirmed these data showing that β-AR blockers prevent AMPHs-induced DA sensitization (Colussi-Mas et al., 2005). These data confirm that DA neurotoxicity, just like autonomic, motor and behavioral effects undergoes sensitization. This is expected since AMPHs-induced sensitization up-regulates those molecular cascades, which are the common pathway to produce all AMPHs-induced alterations.

Similarly, marked alterations in AMPHs-induced DA release within the dorsal striatum occur following a damage to LC *via* DSP-4 and following either genetic or pharmacological blockade of NE synthesis (Weinshenker et al., 2008). Such a neurochemical effect mediated by NE loss enhances the behavioral response induced by METH while potentiating nigrostriatal METH toxicity (Weinshenker et al., 2000, 2008). In addition, a reduction of TH within LC neurons (*via* RNA interference) potentiates D1 receptor-dependent AMPHs-induced sensitization in the ventral striatum, to an extent, which is not replicated by DAT inhibition (Smith and Greene, 2012). In line with this, Harro et al. (2000) found that a loss of NE axons increases AMPHs-induced locomotor activity while up-regulating striatal D2 receptors. Again, a damage to LC neurons enhances nigrostriatal METH toxicity in both mice and rats (Fornai et al., 1995, 1996a,b), an effect, which is related to increased DA sensitivity to METH rather than to METH pharmacokinetics (Fornai et al., 1997, 1998, 1999). Such a potentiation is suddenly evident by observing METH-induced behavioral changes when dramatic stereotypies occur in LC-damaged mice. It is likely that NE operates at some level within DA neurons to alter synaptic plasticity. In fact, the abnormal synaptic plasticity, which happens following pulsatile DA stimulation in a parkinsonian striatum, is worsened in LC-damaged mice, which develop severe abnormal involuntary movements following low doses of L-DOPA (Fulceri et al., 2007). These effects appear to rely more on LC compared with other NE nuclei. In fact, they can be reproduced by a bilateral stereotactic injection of the neurotoxin 6-OHDA within both LC nuclei (Fulceri et al., 2007).

## Reticular Nuclei Within the Dorsal Raphe/PAG as a Paradigm to Decipher AMPH-Induced Behavior

**Figure 7:**
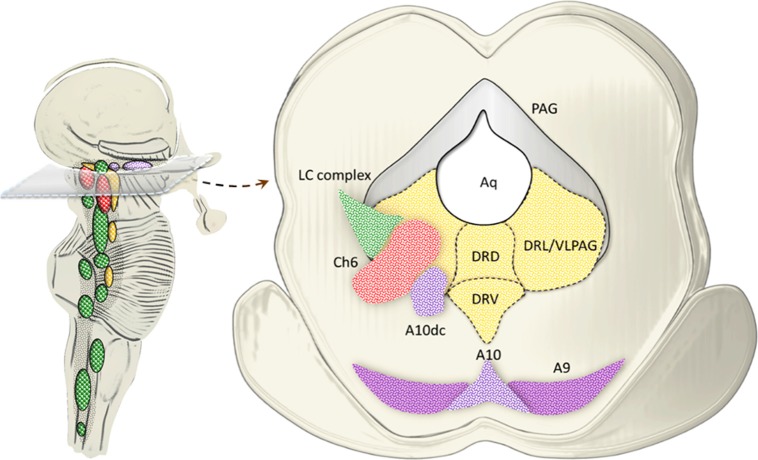
Catecholamine and cholinergic nuclei of the dorsal raphe/PAG. The dorsal raphe nucleus extends from the rostral pons up to the midbrain within and around the ventromedial and ventrolateral PAG. In fact, at this level, three sub-regions of the dorsal raphe intermingling with PAG are identified, namely, dorsal (DRD), ventral (DRV) and lateral (DRL; Steinbusch et al., 1981; Baker et al., 1990, 1991). While 5-HT neurons are scattered throughout all the dorsal raphe, the ventromedial and mostly the ventrolateral PAG (VLPAG) hosts a mixed neuronal population, among which catecholamine and cholinergic neurons prevail at large. This is the case of the NE nucleus epicoeruleus corresponding to the rostral and dorsal extent of the LC complex. Similarly, DA neurons of the A10dc nucleus, as well as cholinergic neurons of the Ch6 (LDTg) intermingle in the DRL/VLPAG with their rostral extent.

Pioneer studies carried out in both animal models and humans uncovered a highly heterogeneous nature of the dorsal raphe neurons, thus providing a seminal contribution in implementing the original description by Dahlstrom and Fuxe (1964). Such a heterogeneity holds true for either cyto-architectural, neurochemical or topographic differences characterizing subsets of neuronal populations within the dorsal raphe. In detail, the dorsal raphe nucleus (B8-B9), which extends from the rostral pons up to the midbrain within and around the ventromedial and ventrolateral PAG, can be subdivided into five sub-regions, namely caudal, dorsal, ventral, ventrolateral and interfascicular (Steinbusch et al., 1981; Baker et al., 1990, 1991). In the present paragraph, we focus on the ventromedial and ventrolateral PAG, where a number of catecholamine nuclei, targeted by AMPHs, are placed. This is the case of the NE nucleus epicoeruleus, DA neurons of the A10dc nucleus, as well as cholinergic neurons of the laterodorsal tegmental nucleus (LDTg, Ch6), which intermingle in the ventral and ventrolateral PAG with their rostral extent (Hökfelt et al., 1984; Mai and Paxinos, 2012; Vasudeva and Waterhouse, 2014; Figure 7).

### AMPHs and Catecholamine Neurons of the PAG

Despite being poorly investigated in the specific case of AMPHs compared with low brainstem reticular nuclei, catecholamine neurons of the PAG represent an important neuro-anatomical substrate for the behavioral changes induced by AMPHs (Tasman and Simon, 1983; Sobieraj et al., 2016). This is not surprising given the plethora of functions, which are regulated by the PAG, such as pain, anxiety, arousal and escape, as well as heart rate, thermogenesis, mean arterial blood pressure and breathing (Bandler et al., 1985; Bandler and Carrive, 1988; Brandao et al., 1990; Carrive, 1991; Lovick, 1993; Coimbra and Brandão, 1997; Hayward et al., 2003). In addition, catecholamine PAG neurons are profusely connected with a variety of cortical and subcortical brain regions. These include, for instance, the thalamus, the medial prefrontal cortex, the basal forebrain cholinergic neurons, the hypothalamic orexin cells, the pontine LDTg, most of the NE bulbo-pontine nuclei, and the VTA (Li et al., 1990; Reichling and Basbaum, 1991; Bajic et al., 2001, 2012; Lu et al., 2006; Rathner and Morrison, 2006). Such a region becomes the prototype for confounding outcomes when trying to decipher the specific effects produced by each monoamine-containing nucleus in AMPHs-induced behavior, which appear largely related to the profuse isodendritic connections occurring within the PAG. For instance, the stimulation of dorsal raphe nucleus, besides enhancing extracellular 5-HT levels in both the forebrain and the LC, also increases extracellular NE (Hajós-Korcsok and Sharp, 2002). Such a response is not altered by a damage to 5-HT neurons of the dorsal raphe. The plethora of connections linking PAG-dorsal raphe neurons with lower NE brainstem nuclei appears critical in AMPHs-induced behavior. In fact, profuse and reciprocal connections occur between PAG and reticular NE nuclei targeted by AMPHs, including LC, A5, A7, PB, and A1/C1 group. For instance, the LC provides a major stimulatory drive to the dorsal raphe nucleus while the A1/C1 provides an inhibitory tone (Peyron et al., 1996; Kim et al., 2004; Cao et al., 2010). In the light of these projections, the PAG becomes an interface in behavioral control concerning the regulation of sleep-wake cycle and arousal, pain modulation and cardiovascular responses (Benarroch, 2012). NE connections with DA-containing nuclei of the PAG are important as well. In fact, following stimulation of LC, α1-AR-dependent NE transmission in the PAG promotes arousal *via* modulation of PAG DA neurons activity (Porter-Stransky et al., 2019). It is remarkable that besides the SNpc, METH targets DA neurons of the PAG, as shown in autopsy brains from METH abusers (Quan et al., 2005). Beyond neurotoxicity, reiterated METH administration induces plastic effects in PAG DA neurons, which associate with drug-induced reward and addiction (Sobieraj et al., 2016). An excitatory effect of TH-positive PAG neurons on the adjacent VTA DA cells (Lu et al., 2006) is likely to participate to AMPHs-induced activation of opioid receptors in the PAG, which associates with analgesia, hyperthermia, and hedonic reward reinforcement (Berridge et al., 2009; Cristina-Silva et al., 2017).

### Acetylcholine-Containing Reticular Nuclei in AMPHs-Induced Behavior

A few studies demonstrated that beyond monoamines, acetylcholine (ACh) is involved in the behavioral effects of AMPHs. In line with this, METH releases ACh in adult mice (Dobbs and Mark, 2008) and alters striatal choline acetyltransferase (ChAT), the enzyme responsible for synthesizing ACh, in humans (Kish et al., 1999; Siegal et al., 2004). Given the critical role of ACh systems in cognition (van Hest et al., 1990; Muir et al., 1994; Lin et al., 1998; Mirza and Stolerman, 2000), alterations in ACh levels and receptors are suggested to contribute to the cognitive impairments observed following METH exposure. Only a few studies investigated the role of ACh produced specifically by reticular brainstem nuclei in AMPH’s-induced effects. The main source of ACh in the brainstem RF is represented by two ACh pontine nuclei, which correspond to the peduncolopontine nucleus (PPN) or peduncolopontine tegmentum (PPTg) and the laterodorsal tegmental nucleus (LDTg). These nuclei are also referred to as Ch5 and Ch6, respectively. Rostrally, the Ch5 is included between two DA nuclei of the RF: ventrally, it contacts the dorsomedial aspect of the A9, while dorsally it is bordered by the A8. Caudally, the Ch5 adjoins the LC. Ch5 neuronal population is heterogeneous regarding its spatial distribution and neurochemistry. In fact, the dorsolateral portion of Ch5, which is called pars compacta, contains densely packed cholinergic neurons forming a continuum with Ch6 cholinergic neurons. Ch6 neurons contour the Ch5 nucleus and rostrally they extend into the PAG and medial longitudinal fasciculus. Caudally to the Ch5 nucleus, Ch6 neurons are intermingled with NE neurons belonging to the epicoeruleus nucleus (Mesulam et al., 1989). Ch6 neurons are slightly smaller than those of Ch5 pars compacta. As components of the RF, Ch5 Ch6 neurons share the typical isodendritic conformation. It is well-established that Ch5 and Ch6 neurons are targeted by the basal ganglia efferent fibers. Projections from these neurons are directed to the thalamus and other nuclei of the brainstem RF (Mesulam et al., 1989). Remarkably, excitatory cholinergic fibers have been described, which project mainly from these two nuclei to the VTA, promoting burst firing in DA neurons and thus enhancing DA release, which is pivotal for AMPHs-induced behavior (Woolf, 1991; Yeomans and Baptista, 1997; Yeomans et al., 2001; Omelchenko and Sesack, 2005, 2006). In particular, Ch6 targets VTA DA neurons, while Ch5 preferentially targets SNpc DA neurons (Oakman et al., 1995). Recent studies suggest that ACh in addition to DA is primarily involved in the initiation and maintenance of hyper-locomotion induced by METH. In detail, Dobbs and Mark (2008) demonstrated that, systemic but not intra-VTA perfusions of METH in mice induce a prolonged ACh release within VTA. In particular, extracellular ACh levels persist above baseline levels for 2–3 h post-injection and it takes 180 and 300 min post-injection to return to baseline values, after a low and high dose of METH respectively. Remarkably, ACh but not DA release, within VTA, correlates dose-dependently with METH-induced locomotor activity. These data suggest that METH acts in the VTA to induce a robust, though short-lived, increase in extracellular DA release while producing a prolonged increase in ACh release, which correlates with hyper-locomotion. In the light of these findings, the same authors investigated the contribution of Ch6 and Ch5 nuclei in METH-induced locomotor activity. Inhibition of ACh release by intra-Ch6 infusion with a muscarinic receptor agonist, which binds to M2 inhibitory auto-receptors, attenuates METH-induced locomotor activity (Dobbs and Mark, 2012). As assessed by brain dialysis, the inhibition of Ch6 neuronal activity blunts METH-induced increase in ACh release within the VTA dose-dependently, while it has no effect on DA release within the NAc.

Ch6 ACh neurons are not involved in METH-induced drug-seeking behavior (Dobbs and Cunningham, 2014), but are important for METH-induced locomotor activity (Dobbs and Mark, 2012). On the other hand, inhibition of ACh release from Ch5 does not produce any effects either on ACh or DA release within VTA and NAc, respectively. Therefore, it is likely that Ch6, rather than Ch5, is involved in locomotor behavior induced by systemic METH, which is mediated by ACh release in the VTA. Previous studies suggest that a damage to both Ch5 and Ch6 blunts hyper-locomotion but enhances stereotypies induced by systemic or intra-ventrolateral striatal injections of AMPHs (Inglis et al., 1994; Allen and Winn, 1995; Forster et al., 2002; Miller et al., 2002). This is due to an increase in DA outflow specifically in the dorsal but not ventral striatum, which suggests that AMPHs-induced hyper-locomotion and stereotypies largely depend on the specific effects of Ch5 and/or Ch6 upon the mesostriatal or mesoaccumbens DA systems. These findings warrant further studies elucidating the site-specificity for ACh in mediating METH-induced behavioral alterations.

Although molecular targets responsible for AMPHs-induced monoamine release are well known, the molecular mechanisms through which AMPHs release ACh are not fully established yet. A stream of interpretation indicates a close functional relationship between DA system and ACh release. This stems from evidence showing that administration of D1 and D2 receptor antagonists blunts AMPHs-induced ACh release within the striatum, hippocampus and frontal cortex (Ajima et al., 1990; Damsma et al., 1990, 1991; Imperato et al., 1993; DeBoer and Abercrombie, 1996; Keys and Mark, 1998). Nonetheless, contradictory results are obtained when a damage to the nigrostriatal DA system is induced by 6-OHDA. In fact, 6-OHDA injections produce only a slight decrease in extracellular ACh levels induced by systemic AMPHs (Mandel et al., 1994; Taguchi et al., 1998). These results led to hypothesize that AMPHs-induced ACh release may be due to connections between cholinergic and catecholamine, rather than solely DA systems. This is supported by evidence indicating that combined administration of the TH inhibitor α-methyl-*p*-tyrosine with the VMAT inhibitor reserpine, completely blocks AMPHs-induced ACh release both *in vivo* and in striatal slices (Cantrill et al., 1983; Taguchi et al., 1998). In any case, once released by AMPHs, ACh provides an important excitatory input to those neurons expressing nicotinic ACh receptors (nACh-Rs). This was mainly investigated on DA neurons, where activation of nACh-Rs leads to intracellular Ca^2+^ accumulation, which in turn facilitates DA exocytosis (MacDermott et al., 1999; Engelman and MacDermott, 2004; Lester et al., 2010). In this way, AMPHs produce DA-related effects also *via* ACh release (Drew et al., 2000; Camarasa et al., 2008; Chipana et al., 2008; Hondebrink et al., 2012). Recently, nACh-Rs-mediated Ca^2+^ increase and subsequent nitric oxide-synthase activation have been linked to AMPHs-induced neurotoxicity (Pubill et al., 2011). In line with this, the pharmacological blockade of α7 nACh-Rs attenuates METH-induced oxidative damage and nigrostriatal neurotoxicity both *in vivo* and striatal synaptosomes. Studies focusing on the effects of AMPHs-induced ACh release on NE system are missing so far, which warrants additional studies to test ACh-NE interplay following AMPHs.

## Author Contributions

MF wrote the article. FL contributed to important intellectual content. FB, CB and LR contributed to the literature review and made artwork. FF was the coordinator of the article. He participated in drafting the article and also in critically revising the article.

## Conflict of Interest Statement

The authors declare that the research was conducted in the absence of any commercial or financial relationships that could be construed as a potential conflict of interest.

## Abbreviations

5-HT: serotonin
6-OHDA: 6-hydroxydopamine
A6sc: nucleus subcoeruleus
Ach: acetylcholine
AMPH(s): amphetamine(s)
AP: area postrema
α1-AR(s): alpha1-adrenergic receptor(s)
α1B-AR: type B alpha1-adrenergic receptor(s)
β-ARs: beta-adrenergic receptor(s)
Cart: cocaine- and amphetamine-regulated transcript
ChAT: choline acetyltransferase
CTZ: chemoreceptor trigger zone
DA: dopamine
DAT: DA transporter
DBH: DA beta-hydroxylase
DMV: dorsal nucleus of the vagus
DNB: dorsal NE bundle
DRD: dorsal dorsal raphe
DRL: lateral dorsal raphe
DRV: ventral dorsal raphe
DSP-4: NE neurotoxin N-(2-chloroethyl)-N-ethyl-2-bromobenzylamine
E: epinephrine
ICSS: intracranial self-stimulation
LC, A6: locus coeruleus
LDTg, Ch6: laterodorsal tegmental nucleus
MAO: monoamine oxidase
METH: methamphetamine
MFB: medial forebrain bundle
NAc: nucleus accumbens
nACh-R(s): nicotinic ACh receptor(s)
NE: norepinephrine
NET: NE transporter
NTS: nucleus of the solitary tract
OX1R: orexin type 1 receptors
PAG: periaqueductal gray
PB: parabrachial nucleus
PKC: protein kinase C
PPN, PPTg, Ch5: peduncolopontine nucleus or tegmentum
RF: reticular formation
RVLM: rostral ventrolateral medulla
SERT: 5-HT transporter
SNpc: Substantia Nigra pars compacta
SPNs: sympathetic pre-ganglionic neurons
VLPAG: ventrolateral PAG
VMAT-2: vesicular monoamine transporter type-2
VNB: ventral NE bundle
VTA, A10: Ventral Tegmental Area.
